# Do β-adrenoreceptor blocking drugs associate with reduced risk of symptomatic osteoarthritis and total joint replacement in the general population? A primary care-based, prospective cohort study using the Clinical Practice Research Datalink

**DOI:** 10.1136/bmjopen-2019-032050

**Published:** 2019-08-01

**Authors:** Georgina Nakafero, Matthew Grainge, Ana Valdes, Nick Townsend, Christian Mallen, Weiya Zhang, Michael Doherty, Mamas A Mamas, Abhishek Abhishek

**Affiliations:** 1 Academic Rheumatology, University of Nottingham, Nottingham, UK; 2 Division of Epidemiology and Public Health, University of Nottingham, Nottingham, UK; 3 Nottingham NIHR BRC, University of Nottingham, Nottingham, UK; 4 University of Bath, Bath, UK; 5 Arthritis Research UK Primary Care Centre, Keele University, Keele, UK; 6 Research Institute for Primary Care and Health Sciences, Keele University, Newcastle, UK

**Keywords:** osteoarthritis, pain, total joint replacement, anti-hypertensives, analgesia

## Abstract

**Introduction:**

To investigate if β-adrenoreceptor blocking drug (β-blocker) prescription reduces the risk of knee or hip osteoarthritis, total joint replacement and analgesic prescription.

**Setting:**

Primary care.

**Methods and analysis:**

This is a cohort study using data from the Clinical Practice Research Datalink. Two separate analyses will be performed. Study 1 will be on the association between β-blocker prescription and incident knee/hip osteoarthritis. Inclusion criteria will be age ≥40 years. Exposed participants will be those with ≥2 continuous β-blocker prescriptions, and the index date will be the date of the first prescription of β-blocker. Unexposed participants will include up to four controls matched for age, sex, general practice surgery and propensity score for β-blocker prescription. Exclusion criteria will include contraindications to β-blockers, consultations for osteoarthritis or potent analgesic prescription before the index date. Outcomes will be knee osteoarthritis (primary outcome), hip osteoarthritis, knee pain and hip pain. Study 2 will be on the association between β-blocker prescription and total joint replacement and analgesic prescription in people with osteoarthritis. Inclusion criteria will be age ≥40 years, knee or hip osteoarthritis, and index date will be as in study 1. Unexposed participants will be as in study 1, additionally matched for consultation for knee or hip osteoarthritis prior to the index date. Exclusion criteria will include contraindications to β-blockers and osteoarthritis in other joints prior to the index date. Outcomes will be total knee replacement (primary outcome), total hip replacement and new analgesic prescription.

**Statistical analysis:**

Kaplan-Meier curves will be plotted, and Cox proportional HRs and 95% CIs will be calculated. Stratified analysis will be performed by class of β-blocker, intrinsic sympathomimetic effect and indication(s) for prescription.

**Ethics and dissemination:**

This study was ethically approved by the Independent Scientific Advisory Committee of the Medicines and Healthcare Authority (Ref 18_227R). The results of this study will be published in peer-reviewed journals and presented at conferences.

**Summary:**

This prospective cohort study will evaluate the analgesic potential of commonly used drugs for osteoarthritis pain.

**Strengths and limitations of this study**

- The study has a large sample size and uses a nationally representative data set.
- The study accounts for propensity score for β-adrenoreceptor drug prescription.
- The study compares the results of propensity score matching and adjustment analysis.

- A limitation is that this is a non-randomised study.
- Data on drug consumption were unavailable.

## Introduction

Osteoarthritis is the most common form of arthritis worldwide. Its prevalence is increasing due to an ageing population and obesity epidemic.^1 2^ It is a leading cause of disability among older adults, with an estimated lifetime risk of 25% and 45% for hip and knee osteoarthritis, respectively.^3 4^ There is no specific therapy for osteoarthritis, and management focuses on symptomatic treatment, with total joint replacement reserved for those with severe symptomatic disease. However, analgesic and anti-inflammatory drugs only have modest efficacy for osteoarthritis pain, with mean Western Ontario and McMaster Universities Arthritis Index improvement of 3.99–11.24 across a spectrum of non-steroidal anti-inflammatory drugs (NSAIDs) and opioids.^5^ Apart from this, NSAIDs and cyclo-oxygenase II inhibitors increase the risk of gastrointestinal bleeding, acute kidney injury, chronic kidney disease, coronary heart disease, hypertension, stroke and congestive cardiac failure, and are associated with increased cardiovascular mortality.^6–18^ These adverse effects are particularly important for people with osteoarthritis as they already have a high burden of cardiovascular comorbidities and risk factors.^19 20^ Consequently, healthcare practitioners are reluctant to prescribe NSAIDs as these patients are often on antiplatelet therapy for primary prevention or dual antiplatelet therapy following a cardiovascular event, meaning that prescription of NSAIDs would expose patients to unacceptably high bleeding risk, and so the prescription of potent opioids for osteoarthritis pain has increased over the last decade.^21^ However, opioids induce dependence and increase the risk of falls, fractures and death.^22–25^ Thus, there is a need to develop safe, effective, well-tolerated, non-addictive analgesics to target osteoarthritis pain that can be prescribed safely to patients with cardiovascular comorbidities.

We recently demonstrated a negative association between self-reported β-adrenoreceptor blocking drug use and pain severity (adjusted OR (aOR) 0.68; 95% CI 0.51 to 0.91) and use of opioid analgesics (aOR 0.73; 95% CI 0.54 to 0.98) in community-dwelling adults with large-joint lower-limb osteoarthritis.^26^ We also found that each year of treatment with β-adrenoreceptor blocking medicines reduces the risk of joint pain (aOR 0.96; 95%CI 0.93 to 0.99; p<0.004),^26^ and that other classes of antihypertensive medications, including α-adrenoreceptor blocking drugs, did not associate with joint pain or analgesic use.^26^

The purpose of this study is to explore the analgesic potential of β-adrenoreceptor blocking drugs and to identify the drug class that is most likely to have an analgesic effect. The specific objectives are to examine the association between β-adrenoreceptor blocking drug prescription and incident knee osteoarthritis, incident hip osteoarthritis, incident knee pain, incident hip pain, analgesic prescription in knee or hip osteoarthritis, and total knee or hip joint replacement. We hypothesise that patients prescribed β-adrenoreceptor blocking drugs are less likely to develop incident symptomatic lower limb osteoarthritis, and in those who have symptomatic osteoarthritis to associate with fewer analgesic prescriptions and to slow the progression to total joint replacement due to the analgesic effect. Additionally, we will also explore data to identify the class of β-adrenoreceptor blocking drug that is most likely to have an analgesic effect.

## Methods

### Study design

This is a cohort study.

#### Data source

Data from the Clinical Practice Research Datalink (CPRD) will be used in this study. Incepted in 1987, the CPRD is a longitudinal anonymised electronic database containing health records of >10 million people in the UK.^27^ CPRD participants are representative of the UK population in terms of age, sex and ethnicity.^27^ The CPRD contains details of diagnoses and primary care prescriptions. The data are enhanced by linkage with hospitalisation (Hospital Episode Statistics) and mortality records (Office of National Statistics).

### Study population

All CPRD registered participants contributing acceptable quality data in up-to-standard general practices (GP) between 1 January 1990 and 31 December 2017 are included. Two separate cohort studies will be performed.

#### Cohort study 1

Cohort study 1 will be on β-adrenoreceptor blocking drug prescription and incident osteoarthritis. Exposure will be ≥2 continuous prescriptions for β-adrenoreceptor blocking drugs, and will be further categorised according to duration of treatment (≤6 months, 7–12 months, 12–24 months and >24 months). The outcomes will be knee osteoarthritis (primary), hip osteoarthritis, knee pain and hip pain.

##### Inclusion criteria

###### Exposed

- ≥2 continuous prescriptions of β-adrenoreceptor blocking drugs defined as two consecutive prescriptions ≤60 days apart.
- Age ≥40 years on the date of the first of the two consecutive β-adrenoreceptor blocking drug prescriptions.

###### Unexposed

- Age (5-year age band based on year of birth), sex, GP surgery, year first registered in CPRD (±1 year) and propensity score for β-adrenoreceptor blocking drug prescription matched to the exposed participants.

##### Propensity for β-adrenoreceptor blocking drug prescription

As participants prescribed β-adrenoreceptor blocking drugs are likely to have comorbidities, be older and have a high body mass index (BMI), a propensity score for β-adrenoreceptor blocking drug prescription will be calculated using a cumulative logit regression model and including demographic factors and relevant diagnoses at any time prior to β-adrenoreceptor blocking drug prescription (box 1).

Box 1 **List of potential variables to be included in the calculation of propensity score for β-adrenoreceptor prescription**

**Demographic factors.**

- Body mass index (WHO classification categories)*, smoking status* and deprivation score (general practice-level Index of Multiple Deprivation Score).

**Comorbidities.**

- Hypertension, angina, myocardial infarction, congestive cardiac failure, atrial fibrillation, stroke, high cholesterol, chronic kidney disease, diabetes, anxiety, migraine, Charlson Comorbidity Index (excluding comorbidities in the propensity score) and duration in years of cardiovascular comorbidities prior to cohort entry.

**Drug prescriptions.**

- Calcium channel blockers, ACE inhibitors, angiotensin II receptor antagonists, bendroflumethiazide, aldosterone antagonists, loop diuretics, alfa-adrenoreceptor blocking drugs, nitrates, nicorandil, aspirin, clopidogrel, statin, fibrate, vitamin K antagonists and non-steroidal anti-inflammatory drugs (in the progression cohort), and as a marker of severity of underlying disease at cohort entry the number of antihypertensive, antiarrhythmic or antianginal drugs prescribed in the 12-month period prior to cohort entry.

*Categorised as missing if not available.

##### Exclusion criteria

Exclusion criteria will be consultation for any of the following prior to the index date:

- Osteoarthritis at any joint.
- Knee, hip, neck or back pain.
- Conditions causing joint damage or chronic pain: autoimmune inflammatory rheumatic diseases, such as rheumatoid arthritis, psoriatic arthritis, ankylosing spondylitis, lupus, polymyalgia rheumatica, gout, radiculopathy, neuropathy and fibromyalgia.
- Contraindications to prescription of β-adrenoreceptor blocking medicines: chronic obstructive pulmonary disease, asthma, peripheral vascular disease, heart block, aortic stenosis and hypertrophic obstructive cardiomyopathy.
- Two consecutive prescriptions for any opioid, any NSAID, gabapentin, pregabalin, duloxetine or amitriptyline prior to the index date.
- <1 year up-to-standard registration prior to index date to reduce the risk of prevalent cases (eg, with osteoarthritis) being classified as incident cases.^28^

The cohort entry date will be the index date. The unexposed participants will be allocated the index date of their matched exposed participant.

The cohort exit date will be the earliest of the date of the last prescription of β-adrenoreceptor blocking drug prescription, plus 4 weeks or a matched pseudo-exit date for the matched controls, date of the first outcome, date of death, transfer-out date, date of the last data collection or study end date (31 December 2017).

#### Cohort study 2

Cohort study 2 will be on β-adrenoreceptor blocking drug prescription and total joint replacement and analgesic use. The exposure will be as in cohort study 1.

The outcome will be total knee replacement (primary), total hip replacement and analgesic prescription: (1) NSAIDs, (2) opioids, (3) gabapentin or pregabalin, and (4) amitriptyline.

##### Inclusion criteria

###### Exposed

- As in cohort study 1 above.
- Consultation for knee or hip osteoarthritis prior to the first of the two prescriptions for β-adrenoreceptor blocking drugs.

###### Unexposed

- As in cohort study 1, but with additional matching for prior consultation for knee or hip osteoarthritis to exposed participants.

##### Propensity for β-adrenoreceptor blocking drug prescription

This will be calculated as in cohort study 1. Additionally, propensity score will include hand osteoarthritis (interphalangeal or thumb base osteoarthritis—a marker of generalised osteoarthritis), neck pain or back pain, spinal degenerative diseases (indicating osteoarthritis in the spine—a marker of generalised osteoarthritis), arthroscopic knee or hip surgery, number of analgesic prescriptions between the first consultation for knee/hip osteoarthritis and β-adrenoreceptor blocking drug prescription, aspirin, statin and bisphosphonate, and glucosamine/chondroitin sulfate prescription in the 12-month period prior to cohort entry, as these may affect knee or hip osteoarthritis progression.^29–33^

##### Exclusion criteria

Exclusion criteria will be previous consultation for any of the following prior to the first prescription of β-adrenoreceptor blocking drug:

- Osteoarthritis at any other joint.
- Neck or back pain.
- Conditions associated with joint damage or chronic pain (as above).
- Conditions contraindicating prescription of β-adrenoreceptor blocking medicines (as above).
- Previous knee or hip replacement.
- Knee or hip joint replacement within 3 months of the date of the first β-adrenoreceptor blocking drug prescription or matched index date in the unexposed participants. These will be excluded because we do not expect β-adrenoreceptor blocking drug prescription to reduce total joint replacement rates immediately and may be commenced during preanaesthetic check-up.
- <1 year up-to-standard registration prior to the first qualifying prescription of β-adrenoreceptor blocking drug.^28^
- Additionally, participants who receive two consecutive prescriptions for opioids, NSAIDs, gabapentin, pregabalin, duloxetine or amitriptyline prior to the index date will be excluded from the cohort when examining the association between β-adrenoreceptor blocking drug prescription and new analgesic prescription.

The cohort entry and exit dates will be as in cohort study 1. Nearest neighbour matching (±0.2 SD) will be used to match exposed to unexposed participants.

### Statistical analysis

Covariate balance between matched exposed and unexposed participants will be examined. Unbalanced covariates will be included in the model as separate covariates. Other covariates that do not influence exposure but influence outcomes or reflect health-seeking behaviour alone may be included in the model after evaluating their balance between exposed and unexposed groups. These are the following:

- Number of GP consultations in the 12-month period preceding cohort entry.
- Number of hospital outpatient referrals in the 12-month period preceding cohort entry.
- Number of hospital admissions in the 12-month period preceding cohort entry.
- Total number of GP consultations for knee or hip injury prior to cohort entry.
- Non-osteoporotic fractures, defined as fractures in any bone except the femur, distal radius and vertebrae, after the age of 18 years but before the age of 50 years in women and 60 years in men. This is a surrogate for physically active occupation or lifestyle that may associate with incident osteoarthritis.

Participants will enter the cohort on the index date, and data will be censored at the earliest of an event, study end date (31 December 2017), date of death, transfer out of CPRD for all outcomes, and additionally date of the last prescription of β-adrenoreceptor blocking drugs, plus 4 weeks (usual duration of GP prescription in the UK) for incident osteoarthritis, incident knee or hip pain, or new analgesic prescription. Given the reported effects of propranolol on pain sensitivity in patients with fibromyalgia,^34^ we anticipate β-adrenoreceptor blocking drugs to have an analgesic effect in the short term, and outcome events >4 weeks after the date of the last β-adrenoreceptor blocking drug prescription will not be included in the analysis, except for total joint replacement which may be delayed due to a period of analgesia.

Time to event data in the exposed and unexposed groups will be compared using Kaplan-Meier survival curves. Schoenfeld residuals will be examined to see if survival curves meet the assumptions of the proportional hazard model. Cox proportional HRs and 95% CI will be calculated for each incident outcome using the first Read code for the event. Similarly, HRs will be calculated for new analgesic prescriptions, defined as prescription for NSAIDs, opioids, gabapentin and pregabalin, respectively, in those exposed to β-adrenoreceptor blocking drugs, with unexposed as the referent.

The analysis will be stratified according to class of β-adrenoreceptor blocking drug used. A likelihood ratio test for interactions will be used to examine the hypothesis that the association between β-adrenoreceptor blocking medicines and incident osteoarthritis differs according to statin, aspirin or bisphosphonate prescription, as their use has been negatively associated with osteoarthritis.

### Sensitivity analyses

β-adrenoreceptor blocking drugs are used first line in the treatment of some conditions, for example, angina (first line according to the National Institute for Health and Care Excellence (NICE) guidelines) and atrial fibrillation,^35^ but may be reserved as add-on therapy for other conditions, for example, hypertension (third or fourth choice drug according to the NICE guidelines). Moreover, the magnitude of the association between indications for prescription of β-blockers and osteoarthritis varies.^36 37^ Therefore, analyses will be restricted to exposed and matched unexposed participants with (1) hypertension, (2) atrial flutter/fibrillation, (3) angina, (4) angina with underlying hypertension or atrial flutter/fibrillation, and (5) congestive cardiac failure. These sensitivity analyses will help account for residual confounding by underlying cardiovascular disease and its severity. Similarly, total joint replacement rates may be affected by the severity of underlying disease for which the β-adrenoreceptor blocker was prescribed.

Further sensitivity analyses will be performed, restricting knee or hip pain as cohort entry criteria in cohort study 2. This is because the Read codes for knee or hip osteoarthritis may only be allocated after several consultations for knee or hip pain. We will repeat our analysis using propensity score for β-adrenoreceptor blocking drug prescription as a covariate to allow us to compare the results from propensity score adjusted and matched analysis.

### Sample size

#### Incident knee osteoarthritis and hip osteoarthritis

The incidence of knee and hip osteoarthritis is 6.5 and 2.1 per 1000 person-years in primary care-based European populations >40 years in age.^1^ Previous research suggests that 6.14% of patients registered in the CPRD are on β-adrenoreceptor blocking drugs, with over two-thirds on treatment for >10 years.^38^ Thus, for a cohort with one exposed and four unexposed participants, the total sample size required to detect an HR of 0.70 for developing knee osteoarthritis and hip osteoarthritis is 88 273 (17 655 exposed) and 273 225 (54 645 exposed) participants, respectively, for a planned study with 80% power and 1% type I error.

#### Progression to joint replacement surgery

The incidence of total knee replacement in community-dwelling adults with knee pain is 9 per 1000 person-years (Dr S Ingham, PhD thesis, University of Nottinghampublished on university website, 2012), and clinical experience suggests that hip osteoarthritis progresses more rapidly (AA, CM, MD). Thus, for a cohort with one exposed and four unexposed participants, the total sample size required to detect an HR of 0.70 for total knee replacement in the exposed participants is 63 753 with 80% power and 1% type I error. As 3.38% of community-dwelling adults with clinically defined knee osteoarthritis are on β-adrenoreceptor blocking drugs (Knee Pain in the Community Study, 2014–2015, Professor Michael Doherty, unpublished data), and given a population prevalence of knee pain of 20% in those over 40s, this gives a potentially eligible exposed population of 40 560 from which to choose 12 755 exposed participants in the CPRD.

#### Plans for addressing missing data

We anticipate that both BMI and smoking will have missing data. Dummy variables will be created to represent missing data for smoking and BMI in the analyses. In addition, multiple imputation sensitivity analyses using chained equations will be carried out to explore any impact that assumptions regarding missing data will have had on any conclusions from the main analyses.

## Discussion

Findings from several studies suggest that β-adrenoreceptor blocking drugs may have an analgesic effect in osteoarthritis. β-adrenoreceptors are distributed widely in the central nervous system, and polymorphisms in β2-adrenoreceptor genes associate with fibromyalgia, chronic widespread pain and irritable bowel syndrome.^39–42^ They have demonstrated analgesic potential in randomised controlled trials in other painful conditions such as fibromyalgia, temporomandibular dysfunction and migraine, and in animal models of arthritis and pain.^34 43–45^ Moreover, β-adrenoreceptor blocking drugs also reduce the need for intraoperative anaesthetics and postoperative analgesia.^46^ This effect has been observed with the highly β1-adrenoreceptor selective drug esmolol, non-β1β2 selective drugs such as propranolol, and drugs such as bupranolol which block the β3-adrenoreceptor.^46^ The mechanism by which β-adrenoreceptor blocking drugs provide analgesia includes heterodimerisation of β2-adrenoreceptors with µ-opioid receptors increasing the latter’s sensitivity,^47^ inhibitory effects on tetrodotoxin-resistant sodium channels in the dorsal root ganglion neurons,^48^ and increased inhibitory neurotransmitter release in the spinal ganglions through a mechanism involving β1-adrenoreceptor-independent Ca^2+^ entry.^45^ If our study demonstrates analgesic potential for β-blockers, we will conduct clinical trials to confirm these findings. If our programme of research demonstrates meaningful analgesic effects of β-blockers for osteoarthritis pain, this class of drugs may be preferred in the treatment of cardiovascular diseases for people with comorbid symptomatic osteoarthritis.

### Patient and public involvement in research

The idea for this study came from people with osteoarthritis who were concerned that the medicines they used for joint pain increased their risk of developing high blood pressure, angina, kidney damage, constipation, falls and so on. This was a serious problem as most of them already had high blood pressure or angina. They expressed the need for a new and safe analgesic that would not cause these side effects. They felt that it would be an additional bonus if the new analgesic could actually be used to treat any coexisting medical conditions, for example, high blood pressure. This unmet need led us to review the scientific literature and perform preliminary research.^26^ The results of this study were discussed at an Arthritis Research UK Pain Centre Patient and Public Involvement (PPI) meeting in July 2016. The PPI volunteers felt that further research is required to confirm the pain-relieving potential of β-blockers. The PPI group felt that the findings should be confirmed in another readily available large database before proceeding to a clinical trial.

### Dissemination

Findings of this study will be disseminated back to the Arthritis Research UK Pain Centre PPI group who helped develop this research. We will also convey the findings to people in the wider community. For this, we will actively engage universities’ existing routes of public communication, including social media engagement. The scientific outcomes of our research will be communicated to the clinical and academic audience via traditional routes of conference papers at national and international conferences and open access research publications in prestigious, widely circulated medical journals.

### Limitations of the study

Drugs available over the counter such as paracetamol are not recorded in the CPRD. It is possible that people on β-adrenoreceptor blocking drugs will consume less over-the-counter NSAIDs and paracetamol. We are unable to measure this as these data are not recorded in the CPRD. As the CPRD does not have data on drug compliance, we cannot account for actual β-adrenoreceptor blocking drug consumption in a dose–response analysis. Similarly, despite our best efforts to minimise confounding, residual confounding may influence the estimates.

## Supplementary Material

Reviewer comments
