# Supplementary material for: Do β-adrenoreceptor blocking drugs associate with reduced risk of symptomatic osteoarthritis and total joint replacement in the general population? A primary care-based, prospective cohort study using the Clinical Practice Research Datalink
Source: BMJ Open. 2019 Aug 1;9(8):e032050. doi: 10.1136/bmjopen-2019-032050 (PMC6688671; doi:10.1136/bmjopen-2019-032050)
Supplement: Reviewer comments [file bmjopen-2019-032050.reviewer_comments.pdf]

PEER REVIEW HISTORY

BMJ Open publishes all reviews undertaken for accepted manuscripts. Reviewers are asked to complete a checklist review form (**http://bmjopen.bmj.com/site/about/resources/checklist.pdf**) and are provided with free text boxes to elaborate on their assessment. These free text comments are reproduced below.

ARTICLE DETAILS

**TITLE (PROVISIONAL)**	Do beta adrenoreceptor blocking drugs associate with reduced risk of symptomatic osteoarthritis and total joint replacement in the general population? A primary care based prospective cohort study using the Clinical Practice Research Datalink.
**AUTHORS**	Nakafero, Georgina; Grainge, Matthew; Valdes, A; Townsend, Nick; Mallen, Christian; Zhang, Weiya; Doherty, Michael; Mamas, Mamas; Abhishek, A

This article was not externally reviewed at BMJ Open. Protocols that have been independently assessed prior to submission to BMJ Open are usually fast-tracked to publication on the grounds that further substantial changes will not be possible. This independent assessment will usually be external, independent review for both a substantial grant award from a non-commercial or government funder and ethics approval.
